# Effect of Bias Voltage on the Microstructure and Photoelectric Properties of W-Doped ZnO Films

**DOI:** 10.3390/nano14242050

**Published:** 2024-12-21

**Authors:** Haijuan Mei, Wanli Wang, Junfeng Zhao, Weilong Zhong, Muyi Qiu, Jiayang Xu, Kailin Gao, Ge Liu, Jianchu Liang, Weiping Gong

**Affiliations:** 1 Guangdong Provincial Key Laboratory of Electronic Functional Materials and Devices, Huizhou University, Huizhou 516007, China; 2 School of Civil Engineering and Architecture, Nanchang Jiaotong University, Nanchang 330100, China

**Keywords:** WZO, bias voltage, microstructure, photoelectric properties

## Abstract

W-doped ZnO (WZO) films were deposited on glass substrates by using RF magnetron sputtering at different substrate bias voltages, and the relationships between microstructure and optical and electrical properties were investigated. The results revealed that the deposition rate of WZO films first decreased from 8.8 to 7.1 nm/min, and then increased to 11.5 nm/min with the increase in bias voltage. After applying a bias voltage to the substrate, the bombardment effect of sputtered ions was enhanced, and the films transformed from a smooth surface into a compact and rough surface. All the films exhibited a hexagonal wurtzite structure with a strong (002) preferred orientation and grew along the c-axis direction. When the bias voltage increased, both the residual stress and lattice parameter of the films gradually increased, and the maximum grain size of 43.4 nm was achieved at −100 V. When the bias voltage was below −300 V, all the films exhibited a high average transmittance of ~90% in the visible light region. As the bias voltage increased, the sheet resistance and resistivity of the films initially decreased and then gradually increased. The highest *F_OM_* of 5.8 × 10^−4^ Ω^−1^ was achieved at −100 V, possessing the best comprehensive photoelectric properties.

## 1. Introduction

Transparent conductive oxide (TCO) films often have a high conductive performance, high transmittance in the visible light wavelength range, and high reflectivity in the near-infrared light wavelength range. Due to its superior photoelectric properties, the TCO film has a broad application value in the field of photoelectric industry [1,2,3]. As the most widely used TCO film, tin-doped indium oxide (ITO) film has the advantages of high conductivity and high transmittance [4,5,6], but the raw material indium is scarce, expensive, and toxic. Reducing or avoiding the consumption of indium has become the inevitable development trend of transparent conductive film. With the development of optoelectronic technology, higher requirements are put forward for the performance of transparent electrode sin flexible displays. As a wide-band-gap semiconductor metal oxide, ZnO film has the advantages of cheap raw materials, rich reserves, and being non-toxic and easy to bend, meaning it is a promising TCO film material. However, the carrier concentration of the undoped ZnO film is not high and the conductivity is poor, meaning appropriate doping is needed to improve the conductivity of the films [7,8].

In recent years, by doping some high-valence elements into ZnO films, more electrons and holes have been obtained to form the N-type semiconductor, thus increasing the carrier concentration and the conductivity of the films, such as Al-doped ZnO (AZO) [9], Ga-doped ZnO (GZO) [10], F-doped ZnO (FZO) [11], Mo-doped ZnO (MZO) [12], and W-doped ZnO (WZO) [13] films. Due to the high valence difference between W^6+^ and Zn^2+^, each W atom can contribute four extra electrons to the electrical conductivity. These additional electrons increase the carrier concentration, thereby reducing the resistivity of the ZnO film and making it more conductive. Moreover, W^6+^ (0.062 nm) has a similar ionic radius to Zn^2+^ (0.074 nm), and it is easy to replace Zn atoms with W without causing significant lattice distortion [14]. Thus, the electrical properties of ZnO films can be effectively improved by doping them with W, as has been widely applied in various fields, including transparent conductive oxides, solar cells, and UV detectors. For example, Abliz et al. [15] deposited WZO films by using radiofrequency (RF) magnetron sputtering, and they found that the appropriate doping of W efficiently reduced the oxygen vacancy defects, carrier concentration, and total trap density, which resulted in good electrical properties and high stability. In addition to element doping, the thickness of WZO films also has a significant impact on its photoelectric properties; for instance, the lowest resistivity together with a high transmittance of 90% were achieved at 332 nm [16]. During the sputtering deposition, process parameters such as substrate temperature [17] and bias voltage [18,19,20,21] have a significant impact on the structure and properties of the films. As an important deposition parameter, the substrate bias voltage is often used to accelerate the sputtered particles to obtain higher energy, promote the migration of atoms on the substrate surface, and help to form a dense microstructure, so as to improve the quality of the films. However, there are few reports on the effect of substrate bias voltage on the photoelectric properties of WZO films.

In this study, W-doped ZnO films were deposited by RF magnetron sputtering at different substrate bias voltages, and the relationships between microstructure and optical and electrical properties were explored.

## 2. Experimental Details

### 2.1. Coating Deposition

WZO films were deposited on ordinary glasses by RF magnetron sputtering using a ceramic target of WZO (99.99% purity, ZnO:W = 98:2 wt%). A rotating substrate holder was placed at the center of the chamber with a rotation speed of 5 rpm, and the distance between the target and the substrate was 115 mm. All the substrates were ultrasonically cleaned in acetone and alcohol for 20 min, respectively, and then placed on a rotating substrate holder subsequent to desiccation. Prior to the deposition, the chamber was pumped to 8.0 × 10^−4^ Pa, and then heated up to 200 °C. High-purity Ar gas was introduced into the chamber, and the flow rate and working pressure were fixed at 80 sccm and 0.5 Pa, respectively. Then, the WZO films were prepared by RF magnetron sputtering for 60 min. During the deposition process, the target power was controlled at 150 W. As the only variable, the substrate bias voltage was varied from 0 to −300 V, and the detailed deposition parameters are shown in Table 1.

### 2.2. Coating Characterization

The surface and cross-section morphologies, as well as thickness of the films were characterized by scanning electron microscopy (SEM, Tescan Vega 3 Xmu, Brno, Czech Republic). The crystalline structure of the films was characterized by X-ray diffraction (XRD, Bruker D8 advance, Karlsruhe, Germany) using Cu *Kα* radiation (λ = 0.15406 nm) in the θ/2θ mode, where the scanning angle and scanning step were 30°–60° and 0.01°, respectively. Based on biaxial strain model analysis [22], Bragg’s law [23], and the Scherrer equation [24], the residual stresses (σ), lattice parameters (c), and grain sizes (D) of films along the c-axis of the (002) plane can be determined, respectively.

(1) $$\sigma =-233\times \frac{c-c_{0}}{c_{0}}$$

(2) $$c=\frac{\lambda }{\sin\theta }$$

(3) $$D=\frac{0.9\lambda }{\beta \cos\theta }$$

where *λ*, *β*, and *c*_0_ = 5.2066 Å refer to the wavelength of the Cu *Kα* radiation, the full width at half maximum (FWHM), and the unstrained lattice constant of ZnO along the c-axis, respectively. The optical transmittance spectrum from 300 to 1000 nm was measured by using a visible spectrophotometer (723PCSR, Ruifeng, Guangzhou, China). The sheet resistance and resistivity of the films were determined by a four-probe resistivity tester (FT-316B, Ruipin Instrument, Ningbo, China).

## 3. Results

### 3.1. Microstructure

**Figure 1:**
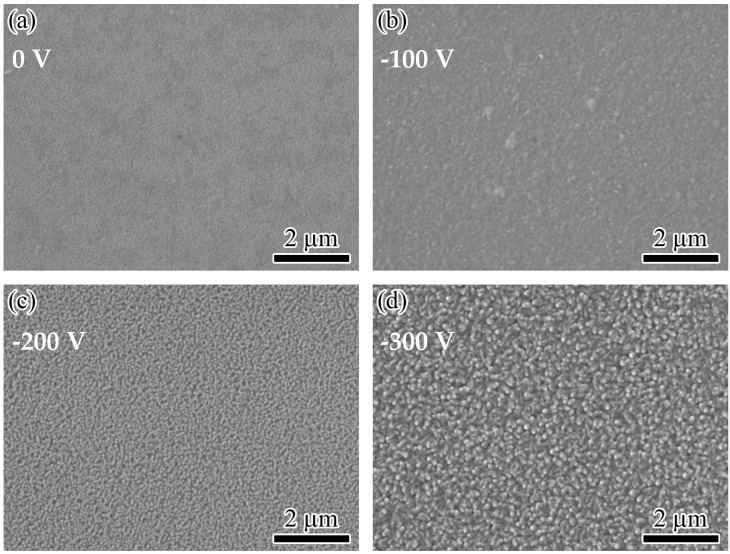
Surface micrographs of the WZO films at various bias voltages: (**a**) 0 V, (**b**) −100 V, (**c**) −200 V, (**d**) −300 V.

Figure 1 presents surface micrographs of WZO films deposited at various bias voltages. As can be seen in Figure 1a, without applying bias voltage, the WZO film showed a compact and smooth surface. After applying a bias voltage of −100 V to the substrate, the surface became rough, accompanied by some large particles, as shown in Figure 1b. When the bias voltage was increased to −200 V, the WZO film showed a compact and rough surface morphology, which was mainly caused by ion bombardment and etching effects under high bias voltage. When the bias voltage was further increased to −300 V, the film presented a rod-like nanocrystal morphology on the surface, which corresponded to the top of the columnar crystal structure of the film. A similar surface structure was also found for the W-doped ZnO films deposited at high substrate temperatures [17]. After applying a bias voltage, the cations in the plasma will continuously bombard the substrate and the growth surface of the film under the action of the electric field, promoting the migration and aggregation of surface atoms and forming large grains. As the bias voltage increased, the ion bombardment effect was further enhanced, causing damage to the growth surface of the films and forming a compact and rough structure.

**Figure 2:**
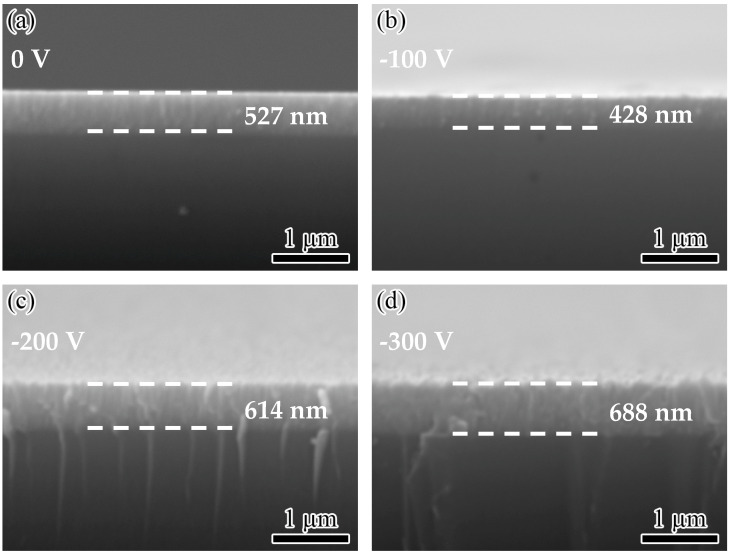
Cross-sectional micrographs of the WZO films at various bias voltages: (**a**) 0 V, (**b**) −100 V, (**c**) −200 V, (**d**) −300 V.

**Figure 3:**
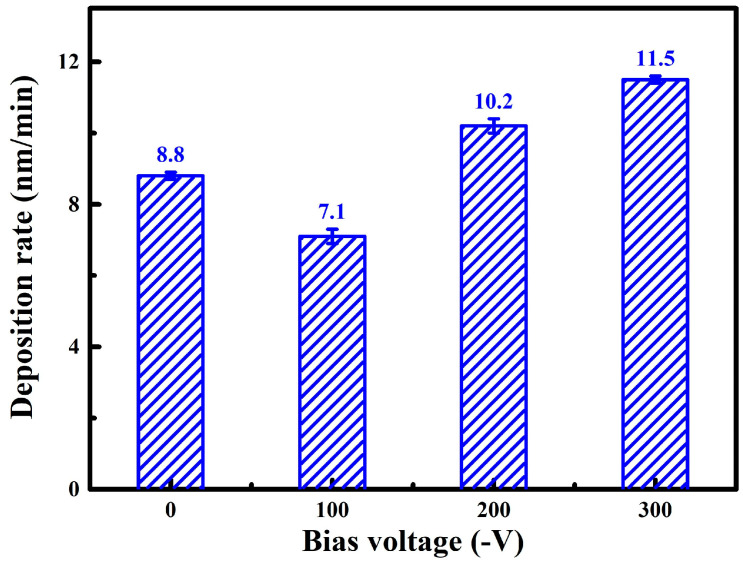
Deposition rates of the WZO films at various bias voltages.

Figure 2 shows the cross-sectional micrographs of WZO films deposited at various bias voltages. As can be seen in Figure 2a, without applying bias voltage, the WZO film showed a fine columnar crystal structure, and the film thickness reached 527 nm. There are no obvious gaps at the interface between the film and the glass substrate, indicating good bonding between the film and the substrate. After applying a bias voltage of −100 V, the WZO film transformed into a dense columnar crystal structure, and the thickness decreased to 428 nm. This can be related to the enhanced ion bombardment effect after applying bias voltage, resulting in the formation of a thin and dense columnar crystal structure. However, when the bias voltage was further increased to −200 and −300 V, the thickness of the films gradually increased, and the films exhibited a distinct columnar crystal structure. As shown in Figure 3, when increasing the bias voltage, the deposition rate of WZO films first decreased from 8.8 to 7.1 nm/min, and then gradually increased to 11.5 nm/min at −300 V. After applying bias voltage, the ion bombardment effect was enhanced, the films became more compact, and this led to an initial decrease in the deposition rate. A similar result was also found for the TiN coatings deposited by magnetron sputtering at various bias voltages [25]. Similarly, the deposition rate of AlTiVCuN coatings initially decreased due to the enhanced ion bombardment effect at high ion source currents [26]. When the substrate bias voltage was further increased, more ions with high energy flew towards the growing surface of the film per unit of time, resulting in an increase in the deposition rate of the films.

**Figure 4:**
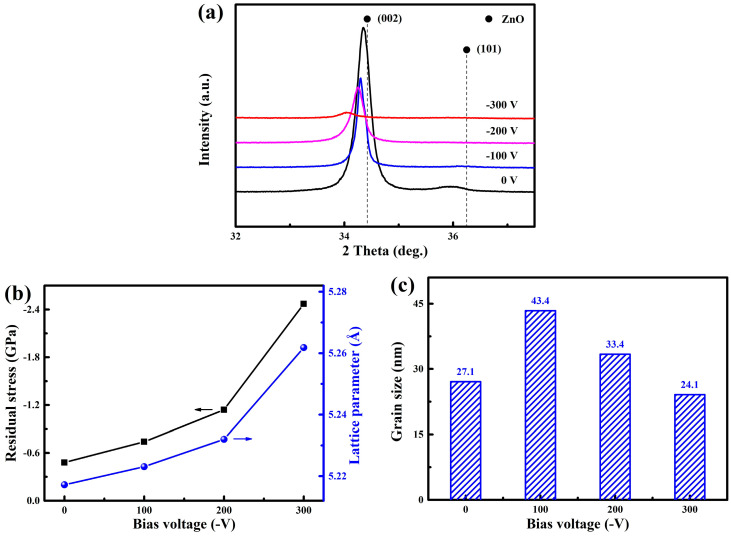
XRD patterns (**a**), residual stresses and lattice parameters (**b**), and grain sizes (**c**) of the WZO films at various bias voltages.

Figure 4a displays the XRD pattern of WZO films deposited at various bias voltages. Without applying bias voltage, a strong diffraction peak appeared at about 34.35°, which corresponded to the (002) plane of the ZnO phase. This indicated that the WZO films showed a hexagonal wurtzite structure with a strong preferred orientation of the (002) plane, that they and grew along the c-axis direction. In addition, a weak diffraction peak appeared at about 35.97°, corresponding to the (101) plane of the ZnO phase, indicating that the WZO films exhibited a polycrystalline structure. However, no W-related phase appeared in the XRD pattern. It was demonstrated by XPS analysis that the W atoms were completely oxidized, and that they existed in the oxidized state of W^6+^ [17]. Due to the similar ionic radii of W^6+^ and Zn^2+^, the doped W tended to replace Zn sites in the hexagonal wurtzite structure [14,15]. When compared to the standard diffraction peak of the ZnO phase (JCPDS 36-1451), both the (002) and (101) diffraction peaks of the films shifted towards lower angles. Generally, due to the difference in ionic radius, replacing Zn^2+^ (0.074 nm) with W^6+^ (0.062 nm) will cause a decrease in the lattice parameters, resulting in a shift of diffraction peaks towards higher angles. Thus, the leftward shift of diffraction peaks was mainly caused by the residual stresses in the films, including thermal stress and intrinsic stress [27]. With the increase in bias voltage, the (002) diffraction peak of WZO films gradually shifted towards lower angles, which was mainly related to the enhanced ion bombardment under higher bias voltages. The calculation of residual stresses and lattice parameters of the films was conducted by Equations (1) and (2), respectively (Figure 4b). All the films exhibited compressive residual stress, and the WZO films presented the lowest compressive residual stress of 0.48 GPa at 0 V. With increasing bias voltage, the residual stress gradually increased to 2.47 GPa at −300 V. Correspondingly, the lattice parameter also gradually increased from 5.2172 to 5.2618 Å. A similar phenomenon was found for the AZO/Cu/AZO films, where the lattice parameter showed a positive correlation with the residual compressive stress [23]. When increasing the bias voltage, the weak (101) peak disappeared, and the intensity of the (002) peak gradually decreased, indicating a decrease in crystallinity of WZO films, which could be due to the increase in lattice defects generated under the strong ion bombardment [28]. In addition, the full width at half-maximum (FWHM) of the (002) peak also varied with the bias voltage. Based on the Scherrer Equation (3), the grain size of the WZO films can be calculated, as shown in Figure 4c. As the bias voltage increased from 0 to −300 V, the grain size first increased from 27.1 to 43.4 nm, then gradually decreased to 24.1 nm, suggesting that the maximum grain size was achieved for the film deposited at −100 V. Under an appropriate bias voltage, the sputtered species with high kinetic energy continuously flew towards the substrate surface, which increased the mobility of surface atoms and then promoted atomic nucleation and growth.

### 3.2. Photoelectric Properties

**Figure 5:**
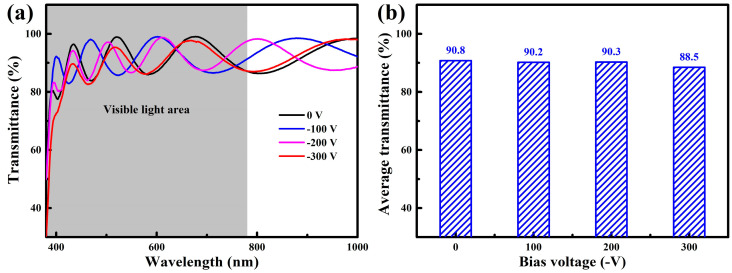
Transmittance (**a**) and average transmittance (**b**) of the WZO films at various bias voltages.

Figure 5a displays the transmission spectra of WZO films deposited at various bias voltages. It can be seen that all the transmission spectra exhibited obvious fluctuations, which can be related to the interference effect caused by the reflection effect at interfaces [16]. Similar fluctuating transmission spectra have also been observed in other W-doped ZnO films [17,29], and the optical band gap of WZO films ranged from 3.45 to 3.52 eV [30]. All the films have a sharp absorption edge in the ultraviolet range of 350–400 nm, and they shifted to a longer wavelength with the increase in bias voltage. Based on the transmittance in the range of 380–780 nm, the average transmittance of the films in the visible light region can be calculated, as shown in Figure 5b. At low bias voltages, all the WZO films exhibited a high average transmittance of ~90% in the visible light region. After applying a high bias voltage of −300 V, the average transmittance of WZO films decreased to 88.5%. This decrease in the average transmittance can be explained by the following reasons. Firstly, with the increase in bias voltage, the plasma bombardment effect was enhanced, resulting in an increase in the surface roughness of the films, which enhanced the scattering of photons, thereby reducing the optical transmittance. Secondly, the film thickness was increased at high bias voltages, which enhanced the absorption of photons, resulting in a decrease in average transmittance [17]. Thirdly, the crystallinity of the film decreased and the grain size became smaller at high bias voltages, resulting in an increase in defects and grain boundaries in the films, which enhanced the scattering effect of photons and thereby reduced the optical transmittance [31].

**Figure 6:**
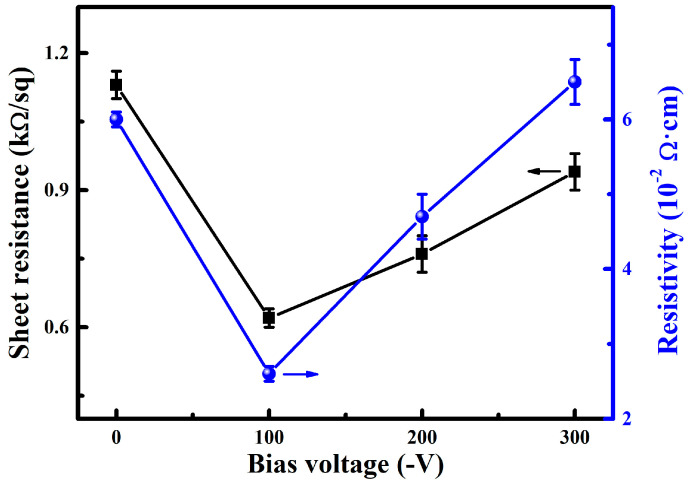
Sheet resistance and resistivity of the WZO films at various bias voltages.

Figure 6 shows the sheet resistance and resistivity of the WZO films deposited at various bias voltages. As the bias voltage increased from 0 to −300 V, the sheet resistance of the films initially decreased from 1.1 to 0.6 kΩ/sq, and then gradually increased to 0.9 kΩ/sq. Correspondingly, the electrical resistivity of the films exhibited a similar trend of change with the increase in bias voltage. Thus, applying an appropriate bias voltage was conductive to improving the electrical conductivity of WZO films, and the lowest electrical resistivity of 2.6 × 10^−2^ Ω·cm was achieved at −100 V, which was lower than that of WZO films with different W doping concentrations [15]. The initially decrease in the sheet resistance and resistivity was mainly attributed to the increase in grain size, as confirmed by the XRD results mentioned above. The larger the grain size, the lower the density of grain boundaries, which reduced the grain boundary scattering and allowed electrons to migrate more freely in the crystal, thereby reducing the electrical resistivity of the films. A similar phenomenon was also found in WZO films deposited at various substrate temperatures, where the electrical resistivity decreased with the increase in grain size [17]. However, when the bias voltage increases above −100 V, the increase in electrical resistivity can be attributed to the poor crystallinity and the smaller grain size. Thin films with low crystallinity usually have more defects, which can capture or scatter free electrons, as well as restrict electron migration, thereby leading to an increase in the electrical resistivity. Similar results were also found in the W-doped ZnO [16] and Zr-doped ZnO [32] films, where the electrical conductivity increased with improved crystallinity.

**Figure 7:**
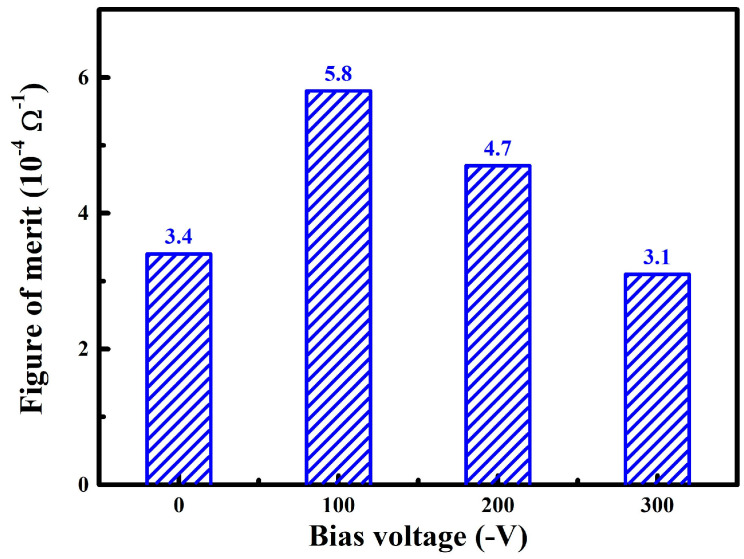
Figures of merit of the WZO films at various bias voltages.

For transparent conductive films, the figure of merit (*F_OM_*) can be used to estimate the comprehensive photoelectric properties, which is defined as follows [33]:

(4) $$F_{OM}=\frac{T_{av}^{10}}{R_{S}}$$

where *T_av_* and *R_s_* refer to the average optical transmittance in the visible region and the sheet resistance, respectively. Figure 7 presents the *F_OM_* of WZO films deposited at various bias voltages. When increasing the bias voltage, the *F_OM_* of WZO films exhibited a trend of first increasing and then decreasing. It can be clearly seen that the highest *F_OM_* of 5.8 × 10^−4^ Ω^−1^ was achieved for the WZO film deposited at −100 V, which possessed a high transmittance of 90.2% as well as a low sheet resistance of 0.6 kΩ/sq. This indicated that the best comprehensive photoelectric properties of the films were achieved at a bias voltage of −100 V. As compared to the WZO films deposited at different substrate temperatures [17], the *F_OM_* was relatively lower, which was mainly related to the high sheet resistance.

## 4. Conclusions

In this study, W-doped ZnO films were deposited on glass substrates by RF magnetron sputtering. The bias voltage was found to have a significant impact on the structure and photoelectric properties of WZO films, providing a simple and effective process solution for preparing high-performance TCO films. The main conclusions are as follows:

(1) When the bias voltage increased from 0 to −300 V, the deposition rate of the films first decreased from 8.8 to 7.1 nm/min and then gradually increased to 11.5 nm/min. After applying bias voltage to the substrate, the ion bombardment effect was enhanced, and the surface morphology became much compact and rough.
(2) All the WZO films exhibited a hexagonal wurtzite structure with a strong (002) preferred orientation, and grew along the c-axis direction. With an increasing bias voltage, the lattice parameter gradually increased from 5.2172 to 5.2618 Å, and the grain size first increased from 27.1 to 43.4 nm and then gradually decreased to 24.1 nm.
(3) All the WZO films exhibited a high average transmittance of ~90% in the visible region. When the bias voltage was increased, the electrical resistivity of the films exhibited a trend of first decreasing and then increasing, and the highest *F_OM_* of 5.8 × 10^−4^ Ω^−1^ was achieved at −100 V.

## Figures and Tables

**Table 1:** Deposition parameters of WZO films.

Parameters	
Base pressure (Pa)	8.0 × 10^−4^
Deposition temperature (°C)	200
Working pressure (Pa)	0.5
Ar flow rate (sccm)	80
Target-to-substrate distance (mm)	115
WZO target	ZnO:W = 98:2 wt%
Target power (W)	150
Substrate bias voltage (V)	0, −100, −200, −300
Deposition time (min)	60

## Data Availability

Data are contained within the article.
